# A Systems Biology Approach to Investigate Kinase Signal Transduction Networks That Are Involved in Triple Negative Breast Cancer Resistance to Cisplatin

**DOI:** 10.3390/jpm12081277

**Published:** 2022-08-03

**Authors:** Nupur Mukherjee, Alacoque Browne, Laura Ivers, Tapesh Santra, Mattia Cremona, Bryan T. Hennessy, Norma O’Donovan, John Crown, Walter Kolch, Dirk Fey, Alex J. Eustace

**Affiliations:** 1 National Institute for Cellular Biotechnology, Dublin City University, D09 NR58 Dublin, Ireland; mukherjeen@nirrh.res.in (N.M.); alacoque.browne@gmail.com (A.B.); laura.ivers@dcu.ie (L.I.); normaodonov@gmail.com (N.O.); john.crown@ccrt.ie (J.C.); 2 Department of Molecular and Cellular Biology, ICMR-National Institute for Research on Reproductive and Child Health, JM Street, Mumbai 400012, India; 3 Systems Biology Ireland, School of Medicine, University College Dublin, D04 E1W1 Dublin, Ireland; tapesh.santra@ucd.ie (T.S.); walter.kolch@ucd.ie (W.K.); 4 Applied Intelligence, Accenture, 1 Grand Canal Square, Grand Canal Dock, D02 P820 Dublin, Ireland; 5 Medical Oncology Laboratory, Smurfit Building, Beaumont Hospital, Royal College of Surgeons in Ireland, D09 YD60 Dublin, Ireland; mattiacremona@rcsi.ie (M.C.); bryanhennessy74@gmail.com (B.T.H.); 6 Department of Medical Oncology, Saint Vincent’s University Hospital, D04 YN63 Dublin, Ireland; 7 Conway Institute, University College Dublin, D04 E1W1 Dublin, Ireland; 8 School of Biotechnology, Dublin City University, D09 NR58 Dublin, Ireland

**Keywords:** triple negative breast cancer, reverse-phase protein array, system biology, BMRA analysis, cisplatin resistance, P53 mutation, PI3K/AKT pathway

## Abstract

Triple negative breast cancer (TNBC) remains a therapeutic challenge due to the lack of targetable genetic alterations and the frequent development of resistance to the standard cisplatin-based chemotherapies. Here, we have taken a systems biology approach to investigate kinase signal transduction networks that are involved in TNBC resistance to cisplatin. Treating a panel of cisplatin-sensitive and cisplatin-resistant TNBC cell lines with a panel of kinase inhibitors allowed us to reconstruct two kinase signalling networks that characterise sensitive and resistant cells. The analysis of these networks suggested that the activation of the PI3K/AKT signalling pathway is critical for cisplatin resistance. Experimental validation of the computational model predictions confirmed that TNBC cell lines with activated PI3K/AKT signalling are sensitive to combinations of cisplatin and PI3K/AKT pathway inhibitors. Thus, our results reveal a new therapeutic approach that is based on identifying targeted therapies that synergise with conventional chemotherapies.

## 1. Introduction

Triple negative breast cancer (TNBC) represents between 10–15% of all breast cancers and can be defined histologically as a subtype of breast cancer that lacks expression of the three key receptors: estrogen, progesterone, and HER2 [1,2]. Without the expression of estrogen, progesterone, and HER2, TNBC lacks a receptor that can be targeted by drugs approved for other subtypes of breast cancer. This lack of classical targets has stimulated much research in an attempt to identify novel targetable drivers of TNBC [3]. The results of these investigations have indicated that TNBC is a very heterogeneous disease which further complicates treatment [3,4]. The main outputs of these research efforts are the addition of immunotherapy, PARP inhibitors in BRCA1/2 mutated TNBCs, and an antibody drug conjugate that targets Trop-2 expressing cells [5]. However, there remain a significant proportion of women to whom no approved targeted therapy is available.

In clinical practice, chemotherapies including platinum-based drugs continue to be the mainstay of standard treatments for TNBC. The use of chemotherapy in TNBC is supported by clinical evidence that demonstrates the benefit of treating breast cancer patients with cisplatin [6]. However, resistance to cisplatin is a significant barrier to long-term disease control [6]. Molecular mechanisms of cisplatin resistance that lead to therapeutic failure and which can also promote tumour growth, progression, and metastasis include abnormal gene expression, reduced intracellular drug accumulation, enhanced DNA damage repair, efficient drug efflux, and altered cell regulatory pathways [7].

In this study, we aimed to reconcile these approaches by testing how cisplatin-based chemotherapy interacts with signal transduction pathways in order to identify targeted therapies that can overcome resistance to cisplatin. Most targeted therapies, such as kinase inhibitors, target components of signal transduction pathways. There is also evidence that DNA-damaging drugs synergize with tyrosine kinase inhibitors (TKIs) that inhibit EGFR to kill TNBC cells [8]. Therefore, we undertook a systematic systems biology approach to investigate whether resistance to cisplatin can be explained and overcome by perturbations of signalling networks that are targetable by TKIs. The Bayesian modular response analysis (BMRA) reconstructed networks revealed significant differences in the wiring of kinase signalling between cisplatin-resistant and cisplatin-sensitive TNBC cell lines. Our analysis showed that the hyperactivation of the PI3K/AKT axis plays a decisive role in cisplatin resistance and can serve as a target that synergises with cisplatin therapies.

## 2. Materials and Methods

### 2.1. Cells and Reagents

TNBC cell lines BT20, HCC1143, MDA-MB-157, MDA-MB-231, MDA-MB-468, MFM-223, and the immortalized mammary epithelial cells MCF10A cells were obtained from the American Tissue Culture Collection (Rockville, MD, USA). TNBC cell lines CAL120, CAL851, and HDQP1 were obtained from the German Tissue Repository DMSZ (Braunschweig, Germany). All cell lines were tested for mycoplasma and authenticated by short tandem repeat (STR) typing. The HCC1143, MDA-MB-231, and MDA-MB-468 cells were cultured in RPMI (Sigma-Aldrich, St. Louis, MO, USA) containing 10% fetal calf serum (FCS; Life Technologies, Carlsbad, CA, USA); the HDQP1 cells were cultured in DMEM (Sigma-Aldrich) containing 10% FCS; the CAL120 and CAL851 cells were cultured in DMEM (Sigma-Aldrich) containing 10% FCS, 1 mM sodium pyruvate, and 2 mM glutamine (Life Technologies); the BT20 cells were cultured in DMEM-HAM F12 (Sigma-Aldrich) containing 10% FCS; the MDA-MB-157 cells were cultured in Leibovitz L15 (Sigma-Aldrich) containing 10% FCS. MCF10A cells were cultured in MEGM with 0.5 mL insulin, 0.5 mL hydrocortisone, 0.5 mL rhEGF and 2 mL BPE. Cells were incubated at 37 °C and 5% CO_2_.

Ipatasertib (S2808), TAK-632 (S7291), PF-00562271 (S2672), Dorsomorphin (S7840), CHIR-98014 (S2745), Everolimus (S1120), PF-4708671 (S2163), and Stattic (S7024) were purchased from SelleckChem, whilst AICAR (Acadesine) (NA03552) and QNZ (EVP4593) (1237744-13-6) were purchased from Carbosynth, and U0126 (V1121) was purchased from Promega.

QNZ (EVP4593), Ipatasertib, Stattic, CHIR-98014, PF-4708671, AICAR (Acadesine), PF-00562271, and U0126 were made up to 10 mM stocks, whilst Dorsomorphin (7.4 mM) and Everolimus (10μM) were also reconstituted in DMSO to the stock concentrations listed. Cisplatin (Selleckchem Cat #S1166 50 mg) was prepared in a stock solution in DMF at a concentration 12 mg/mL (39.99 mM).

### 2.2. Proliferation Assays

A total of 5 × 10^3^ cells/well for MDA-MB-157 cells, 4 × 10^3^ cells/well for CAL851 cells, and 3 × 10^3^ cells/well for the other cell lines were seeded in 96-well plates. Following overnight incubation at 37 °C, drugs were added at the indicated concentrations and incubated for 5 days at 37 °C. Cell proliferation was determined using the acid phosphatase assay, as described previously [9]. Inhibition of proliferation was calculated relative to untreated controls. The effective dose of drug that inhibits 50% of growth (IC_50_ values) and combination index (CI) values were determined using the Chou–Talalay equation on CalcuSyn software. Loewe synergy was calculated using Combifit Analysis ™ [10].

### 2.3. Determination of Baseline Protein Expression

On day 0 of the experiment, MDA-MB-231 cells were seeded at 0.45 × 10^6^ cells per well whilst CAL851, MCF10A, and HDQP1 cells were seeded at 0.3 × 10^6^ cells per well, and MFM-223, MDA-MB-468, and MDAMB-157 cells were seeded at 0.5 × 10^6^ cells per well in 6 well plates. Cell density was chosen to reflect 75–80% confluence prior to starting treatment. Afterward, cells had the attached media removed and replaced with serum-free medium at least 16 h prior to treatment with inhibitors.

On day 1, cell lines were treated with the relevant drug as per Table 1 for a duration of 1 h. Drug-containing media was then removed from the cells and the cells were washed twice in ice cold PBS; 50 μL of RPPA lysis buffer (1% Triton X-100, 50 mM HEPES pH 7.4, 150 mM NaCl, 1.5 mM MgCl2, 1 mM EGTA, 100 mM NaF, 10 mM sodium pyrophosphate tetrabasic, 1 mM sodium orthovanadate, 10% glycerol) containing 7× cOmplete protease inhibitors (Roche, Cat. #05056489001), and 10X phosSTOP Phosphatase inhibitors (Roche, Cat. #04906837001) was added to the cells and the cells incubated on ice for 20 min. Cells were scraped and the lysate collected into an Eppendorf. To aid with the lysis of the cells, Eppendorfs were placed on a shaker at 4 °C for 20 min and the resulting lysate was spun at 14,000 RPM for 10 min at 4 °C. Quadruplet samples were prepared for RPPA experiments.

Protein quantification was performed on samples using the bicinchoninic acid assay (Pierce Biotechnology) and subsequently stored at −80 °C until required.

Samples were prepared for RPPA analysis by adjusting protein concentrations to 1 μg/μL and by the addition of 4× SDS Sample buffer (40% glycerol, 8% SDS, 0.25 Mm Tris-HCL (pH 6.8) +1/10 Bond breaker TCEP solution (Pierce, Cat +77720). Samples were heated to 96 °C for 5 min and the prepared sample was collected and stored at −80 °C until further analysis.

### 2.4. Reverse-Phase Protein Array

Baseline and on-treatment determination of proteins/phosphorylated proteins in the panel of 6 TNBC cell lines and the MCF10A cell line were determined by RPPA as described previously [11]. The antibodies used are listed in Supplementary Materials and Methods.

### 2.5. Bayesian Molecular Response Analysis (BMRA)

Network inference was performed for each cell line using the corresponding perturbation data by applying the BMRA algorithm described previously, and available as MATLAB code [12]. The prior network included all known canonical interactions (Supplementary Figure S2A), and interactions from all perturbed nodes to all other nodes with two exceptions: Raf-only phosphorylated MEK, and MEK-only phosphorylated ERK. BMRA relies on the assumption of signalling modules interconnected by information flow. Consequently, analytes that measure different phosphorylation on the same protein belong to the same module and were lumped into one node by averaging their measured values. The following nodes were lumped: AKT_S473_ and AKT_T308_ into pAKT; mTOR_s2481_ and mTOR_S2448_ into mTOR_p; S6RIB_S235_ and S6RIB_S240_ into S6RIB_p. The following 18 nodes were considered for BMRA network inference: cRAF_S338_, MEK1_2_S217_, MAPK_T202_, PI3KalPha, PTEN, PDK1_S241_, AKT_p, mTOR_p, GSK3B_S9_, AMPK_T172_, P70_S6K_T389_, S6RIB_p, NF_kB_p65, CHK1_S345_, p38MAPK_T180_, p53, stat3_y750_, FAK_Y925. BMRA was run using the following hyperparameters: number of iterations noit = 1000; burnin = 500; times = 10,000. The analysis was implemented in MATLAB R2019b and the code for the computational analysis is available in github: (https://github.com/dirkfey/TNBC-RPPA accessed on 15 February 2022).

### 2.6. Clustering

Hierarchical clustering using Euclidean distance and average linkage was performed using the MATLAB R2019b clustergram function.

### 2.7. Statistical Analysis

Spearman’s rank correlation was conducted using the PRISM software package, and a *p*-value < 0.05 was considered significant.

## 3. Results

### 3.1. Classification of Cisplatin Response in a Panel of TNBC Cell Lines

We tested the antiproliferative effects of cisplatin in a panel of eight TNBC cell lines representing the main subtypes of TNBC (Table 2). To define a cutoff for cisplatin sensitivity/resistance, we also determined the IC_50_ to cisplatin in the immortalised normal breast epithelial cell line MCF10A (5.25 ± 0.55 µM). Taking their IC_50_ as a guide to avoid harming normal epithelial tissue, we categorised our panel of TNBC cell lines as cisplatin-sensitive if they had an IC_50_ of < 5 µM, or cisplatin-resistant if they had an IC_50_ to cisplatin of >5 µM. We also classified cisplatin response in our panel of TNBC cell lines based on their molecular subtype, as determined by Lehmann et al. [1], but found no correlation.

We utilised a systems biology approach with reverse protein array data to determine inferred networks that predict inhibitor sensitivity. We performed RPPA analysis on a panel of seven TNBC cell lines that included three cisplatin-resistant and three cisplatin-sensitive cell lines and the normal MCF10A cell line. We tested a panel of 11 inhibitors that target nodes of pathways found altered in breast cancer, including the AMPK, STAT, NFkB, PI3K/AKT, and MAPK/ERK pathways in each cell line. The RPPA contained a panel of 72 proteins, which was enriched for members of the PI3K/AKT and MAPK pathway to determine differences in protein expression and phosphorylation between untreated and inhibitor-treated cell lines.

First, we wanted to understand if there were any drug-specific response patterns across the cell lines. Thus, we clustered the treatment-induced fold changes for each drug across the seven cell lines (Supplementary Figure S1). A few observations could be made. Firstly, four drugs had little effect on the measured network nodes with less than 10 nodes significantly changing in response to the drug (Supplementary Figure S1A), while the number of changes for the other drugs ranged from 9 for U0126 (and 27 for Everolimus) to 83 for AICAR. Drugs with less than 10 changes were cisplatin with four changes, and the targeted inhibitors of FAK (PF-00562271) and p70S6K (PF-4708671) with 5 and 9 changes, respectively (Supplementary Figure S1A). Secondly, the noncancerous MCF10A cell line was the most responsive cell line (Supplementary Figure S1B), with both the highest number of nodes changing per treatment and the highest average fold-change per node (absolute log2 fold change of 1.2 for MCF10A versus <0.3 for all other cell lines). Thirdly, five drugs affected more than 50 network nodes and exhibited similar response patterns. These were the AMPK activator AICAR, the ALK2/3/6 and AMPK inhibitor Dorsomorphin, the GSK3B inhibitor CHIR-98014, the NFkB inhibitor QNZ, and the RAF inhibitor TAK-632 (Supplementary Figure S1A; top panels). Lastly, the AKT inhibitor ipatasertib caused a strong upregulation of AKT phosphorylation, indicative of the release of a negative feedback loop (Supplementary Figure S1A; panel ipatasertib). The observed upregulation of AKT phosphorylation at the AKT-T308 residue was greater than at the AKT-S473 residue, but only in sensitive cell lines (Supplementary Figure S1A; panel ipatasertib). This suggests an impaired negative feedback from mTORC1 to AKT in resistant cell lines.

Next, we identified the cell-line specific interaction networks from these data using Bayesian molecular response analysis (BMRA) [12]. BMRA uses the inhibitor-induced fold changes of all network nodes and drugs to compute the underlying network interactions that can best explain the data. The advantage of modular response analysis (in contrast to other correlation-based network analyses) is that it can infer causal relationships between the measured network nodes, provided that each network node has been perturbed one-by-one when generating the perturbation data. BMRA lifts this limitation by being able to work with datasets in which a limited number of nodes were perturbed. Consequently, our RPPA dataset is ideally suited for causal network inference using BMRA.

**Figure 1:**
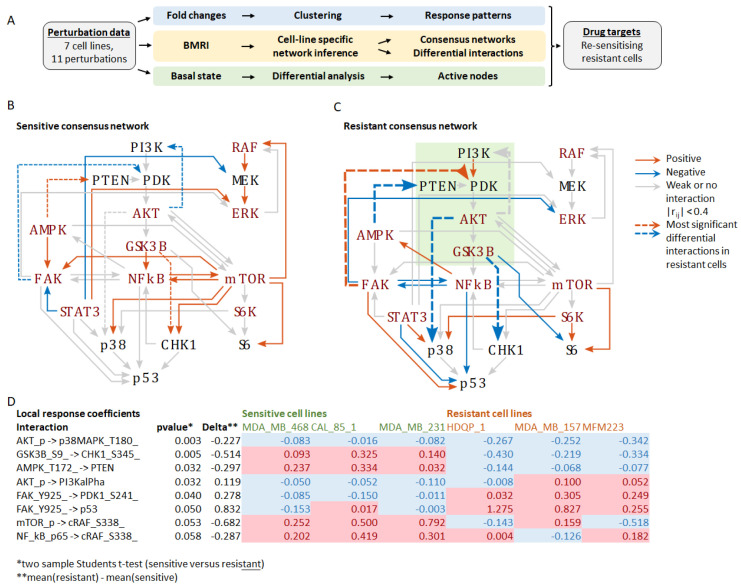
Inferred networks based on the perturbation experiments. (**A**) Schematic overview of the workflow depicting the three analyses performed: (1) Fold-change analysis in response to the treatments, (2) BMRI: Bayesian modular response analysis for network reconstruction, (3) Differential analysis of the basal (untreated) network state to identify the most active nodes in resistant versus sensitive cell lines. (**B**) Consensus network of the sensitive cell lines. (**C**) Consensus network of the resistant cell lines. Edges represent the BMRA-inferred network interactions (from Supplementary Figure S2) averaged over the sensitive and resistant cell lines as indicated in the legend. Strong interactions with local response coefficients greater that 0.4 are indicated with blue (negative) and red (positive) solid lines. Dashed lines indicate the most significant differential interactions in resistant cell lines (see (**D**)). The green box indicates the PI3K-AKT signalling axis. (**D**) Table of the most significant interaction differences between sensitive and resistant cell lines. Negative values shaded blue indicate inhibition; positive values shaded red indicate activation.

To identify critical differences in network connectivity and interaction strength in resistant versus sensitive cells, we applied BMRA to the drug perturbation data from each cell line separately (Supplementary Figure S2A). Focusing on the measured phosphoproteins, but also including PTEN and p53, the BMRA network consisted of 18 nodes (see Materials and Methods), with cell-line specific interactions inferred by the algorithm. The number of inferred interactions by each cell line was: MDA-MB-468, 91; CAL-85-1, 110; MDA-MB-231, 98; MCF10A, 93; HDQP-1, 95; MDA-MB-157, 115; and MFM223, 94. The cell-line specific interaction networks, including the numbers of incoming and outgoing interactions per node, are reported in Supplementary Figure S2A. These BMRA-inferred interactions constitute the networks that best explain the experimental perturbation data. Then, two consensus networks were constructed by keeping only those interactions that were either all positive or all negative across the sensitive and resistant cell lines and averaging their interactions strength (Supplementary Figure S2B). The results show widespread rewiring of the network in resistant cell lines with several feedbacks and cross-talks changing the sign of the interaction (Figure 1). The most significant changes (*p*-value < 0.05, two-sample students t-test) in the resistant versus sensitive network were (1) markedly increased inhibition of p38 MAPK by AKT, (2) change from activation to inhibition of CHK1 by GSK3B, (3) change from activation to inhibition of PTEN by AMPK, (4) a loss of negative feedback from AKT to PI3K, and (5) a change from inhibition to activation of PDK1 by FAK (Supplementary Figure S2C, Figure 1). These changes should activate the PI3K-AKT signalling axis by suppressing the negative regulator PTEN and stimulating the upstream PI3K activator PDK1. They also rewire the downstream connections of the PI3K-AKT module, resulting in inhibition of the stress-activated kinase p38 and the DNA damage response kinase CHK1. Interestingly, these kinases cooperate during the DNA damage response with CHK1 being required for implementing the G2 DNA damage checkpoint and p38 promoting apoptosis [13,14]. These finding suggest that the activation and rewiring of the PI3K-AKT signalling axis is critical for cisplatin resistance and a potential target for drug combinations to overcome cisplatin resistance.

To constitute a good/promising drug target, the targeted network node(s) should be active in the basal state. Thus, we assessed the networks’ basal activity states in the unperturbed condition (Supplementary Figure S2D). Several network nodes related to the PI3K-AKT signalling axis showed increased activity in resistant cell lines, including PI3Kalpha, PDK1, AKT-S473, while the AKT substrate p27^kip^ was phosphorylated and inactivated. Therefore, we can conclude that the PI3K-AKT signalling axis is constitutively active in resistant cell lines and has promise as a drug target.

To check whether PI3K, PDK1, and AKT are expressed in triple negative breast cancer and whether this expression correlates with relapse-free patient survival, we analysed data from the METABRIC study. In triple negative patients (*n* = 299), AKT1 mRNA expression was downregulated compared to both HER2 positive $p=2.2\cdot 10^{-16}$, *n* = 127) and hormone receptor positive patients ($p=9.9\cdot 10^{-10}$, *n* = 1369, Supplementary Figure S2E), whereas PIK3CA was upregulated (p=0.0085 and $p=2.2\cdot 10^{-7}$, Supplementary Figure S2F), and PDK1 showed no significant change with respect to HER2 status and was downregulated in comparison to hormone receptor positive patients ($p=7.5\cdot 10^{-10}$, Supplementary Figure S2G). Increased expression of AKT1 (*p* = 0.00023, *n* = 299 patients), but not PIK3CA (*p* = 0.032 > 0.01) and PDPK1 (*p* = 0.14), significantly associated with poor relapse-free survival in triple negative patients (Supplementary Figure S2H–J).

In summary, our network analysis suggests that PI3K, PDK1, and AKT might be promising combination drug targets to overcome cisplatin resistance because the PI3K-AKT signalling axis (i) is upregulated in resistant cell lines, (ii) features several crosstalk and feedbacks that are more active in resistant cell lines, (iii) overrides DNA damage checkpoints by inhibiting CHK1, (iv) enhances survival by suppressing p38 activity, and (v) promotes cell proliferation by inactivating the cell-cycle inhibitor p27kip [15].

### 3.2. Combination of PI3K/AKT Inhibitors and Cisplatin Are Effective in Double TP53/PIK3CA Mutant TNBC

The results of this computational analysis reverberate with biological findings. PTEN and PI3KCA (the catalytic subunit of PI3K) are often mutated in TNBC resulting in the constitutive activation of the PI3K-AKT pathway [16]. However, in our study PIK3CA mutational status of the TNBC cell lines does not correlate with cisplatin response. Moreover, TNBC features frequent TP53 mutations, and previous studies have linked TP53 mutation status with sensitivity to PI3K inhibitors. Therefore, we classified the PIK3CA and TP53 mutation status for each of the nine TNBC cell lines using the CCLE website (Table 3). Analysis of a subset of patients from the METABRIC dataset who were negative for receptor expression (HER2-/ER-/PR-, *n* = 237) showed that TP53 mutations occur in 81.9% of TNBC patients whilst PIK3CA mutations occur in 14.8% TNBC patients. Further, we determined that PIK3CA and TP53 mutations are neither mutually exclusive nor do they significantly co-occur in TNBC patients [17,18], suggesting that they are functionally independent of each other. Our analysis found that PIK3CA mutations occurred in 2/6 cisplatin-sensitive cell lines and in 1/3 cisplatin-resistant cell lines, whilst only 1/9 cell lines was TP53 WT. Owing to TP53 mutations being overrepresented in our TNBC models, we then aimed to determine the impact of co-occurring TP53/PIK3CA mutations on response to dual targeting with a PI3Ki and cisplatin.

Therefore, we focused our investigation on possible links between TP53 and PIK3CA mutational status and cisplatin response in TNBC cell lines, which were treated with cisplatin and a PI3K/AKT pathway inhibitor. For this, we tested inhibitors for multiple points along the PI3K/AKT/S6-kinase axis that were identified from our systems biology analysis (Table 3). The S6-kinase inhibitor (M2698) had anti-proliferative effects in our panel, with IC_50_s ranging from 0.11 ± 0.02 μM in MFM-223 cells to 7.34 ± 3.40 μM in HDQ-P1 cells. We correlated sensitivity of the S6-kinase inhibitor with cisplatin and found no significant correlation (Spearman rank correlation r = 0.2143, *p* = 0.6615). We further assessed the GSK3B inhibitor CHIR-98014 in our panel of TNBC cell lines, but found limited antiproliferative response with only one cell line achieving an IC_50_ at 1 μM (MDAMB-468). Lastly, we tested the PI3K (αδ specific) inhibitor taselisib and the AKT inhibitor ipatasertib in a panel of TNBC cell lines. As expected, cell lines with PIK3CA mutations were most sensitive to the PI3K/AKT inhibitors.

**Figure 2:**
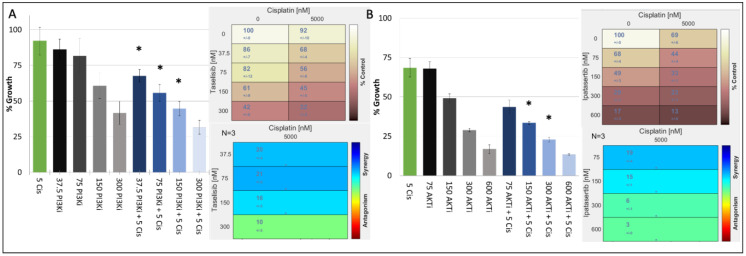
Growth inhibitory effect of cisplatin (μM) in combination with either (**A**) taselisib (PI3K inhibitor) (nM) or (**B**) ipatasertib (AKT inhibitor) (nM) in the MFM223 breast cancer cell line (TP53 Mutant/PIK3CA Mutant). Standard deviations are calculated from triplicate independent assays. Statistical analysis conducted using Prism, where a *p*-value of <0.05 was deemed statistically significant using a repeated measures ANOVA, which was corrected using Tukey’s multiple comparison test. Significant results are indicated with ‘*’. (**A**) Combifit analysis calculates the average % growth of either cisplatin or taselisib tested alone or in combination, whilst Loewe analysis calculates the synergy of the combination. (**B**) Combifit analysis calculates the average % growth of either cisplatin or ipatasertib tested alone or in combination, whilst Loewe analysis calculates the synergy of the combination cisplatin and ipatasertib tested alone or in combination.

In our TNBC cell-line panel, we demonstrated that the proliferation of cisplatin responsive TNBC cell lines with or without TP53 mutations could be decreased by inhibiting key nodes of the PI3K/AKT S6 kinase pathway. We therefore determined the antiproliferative effects of combining a PI3K inhibitor (taselisib) or the AKT inhibitor ipatasertib with cisplatin in a panel of cisplatin-sensitive/resistant TNBC cell lines (Table 4). The results showed that cisplatin and PI3K inhibitors synergised regardless of the cells’ inherent cisplatin sensitivity. The cell line most sensitive to the synergistic combination of PI3K inhibitors and cisplatin was the cisplatin-resistant, PIK3CA Mut/TP53 Mut cell line, MFM-223 (Table 4, Figure 2A).

When we analysed the synergism to PI3K inhibitor and cisplatin in the remaining cisplatin-resistant cell lines, we found that the combination showed limited synergy in cell lines that harboured TP53 mutation but were PIK3CA wildtype (WT) (HDQP1, MDAMB157). We also found that the CAL-51 cell line which is cisplatin sensitive and PIK3CA WT but harbours a TP53 mutation showed reduced synergy to the combination of cisplatin and a PI3K inhibitor. Finally, the BT20 cell line which is cisplatin sensitive but has mutations in both PIK3CA and TP53 exhibited strong synergism to the combination of cisplatin and PI3K inhibition. However, the strongest synergism between cisplatin and PI3K was observed in MFM223 cells. To test whether this synergy extends down the pathway, we combined an AKT inhibitor with cisplatin, also observing a strong synergy (Table 4, Figure 2B).

Overall, our analysis suggests that cisplatin-resistant TNBC cell lines can be resensitised to cisplatin by combination therapies with either a PI3K inhibitor or AKT inhibitor, if they also harbour mutations in PIKC3A and TP53.

## 4. Discussion

Chemotherapies that target DNA including anthracycline- and platinum-based drugs contribute towards the standard treatments for many cancers [7]. In TNBC, platinum-based drugs such as carboplatin/cisplatin- or anthracycline-based drugs including epirubicin have been used alone or in combination to treat patients [19]. Further, clinical evidence has demonstrated the benefit of treating breast cancer patients with cisplatin [6,7], and in TNBC, cisplatin combined with targeted therapies or other chemotherapies results in better response rates [20,21] when compared to platinum-based treatment alone.

Despite these advances, cisplatin’s efficacy to treat cancer can be limited due to either acquired or intrinsic resistance—a clinical observation also observed in TNBC [7]. Resistance and the toxicity associated with cisplatin treatment [7] remains a significant problem in identifying which TNBC patients will respond best to its use. Mechanisms of intrinsic resistance to cisplatin in cancer include the increased expression of efflux pumps associated with multidrug resistance (MDR) [7,22,23,24]. Despite recent publications that address the advances in systems biology approaches to understanding the mechanism of intrinsic cisplatin resistance [22,23,25], our study is the first to utilise a proteomics-based (reverse-phase protein array—RPPA) network modelling approach to identify novel pathways and treatments that can overcome innate cisplatin resistance.

We utilised RPPA data and applied BMRA to reconstruct signalling pathways and their rewiring in cisplatin-sensitive and cisplatin-resistant cell lines. It is important to acknowledge that the TKIs used in this study—similar to all kinase inhibitors—may have off-target effects. However, it is not currently possible to determine every single off-target effect of specific TKIs [26]. Therefore, we prioritised those targets for which the TKI was designed. As the response to drugs is dynamic, we employed BMRA, a method that can faithfully reconstruct signal transduction networks from dynamic responses to targeted drugs [12] in cisplatin-sensitive and cisplatin-resistant TNBC cell lines. The advantage of BMRA is that it can reconstruct causal relationships between network nodes, in this case proteins, and also quantitatively determine whether a node activates or inhibits other nodes. This capability enables us to perform a network-level analysis of drug resistance and identify potential mechanisms how to overcome it by considering changes in the whole network as targets rather than single proteins.

The BMRA analysis reconstructed two consensus networks, one from cisplatin-sensitive and the other from cisplatin-resistant cell lines showing that a large number of connections were rewired either qualitatively or quantitatively. Looking at how the drug-resistant network could be converted into a sensitive configuration initially suggested that cisplatin-resistant TNBC cell lines should have greater sensitivity to inhibition of either p70S6-kinase or GSK3B. However, in our panel of TNBC cell-line sensitivity to either p70S6-kinase (M2698) or GSK3B (CHIR-98104) did not correlate with cisplatin resistance. We showed that the MFM223 cell line which is cisplatin resistant is the most sensitive to M2698, and previous studies also displayed M2698 effectively inhibited in vivo TNBC tumour growth. However this antitumour effect could be associated with cell lines that harboured PIK3CA mutations [27], such as the MFM223 cell line.

It is known that PTEN and PI3KCA (the catalytic subunit of PI3K) are often mutated in TNBC, resulting in the constitutive activation of the PI3K-AKT pathway [16]. The frequency of mutations in either PIK3CA and or AKT that lead to PI3K-AKT pathway activation may occur in up to 30% of TNBC [16], and targeting the PI3K/AKT pathway has been tested clinically in TNBC [28]. Supporting our hypothesis of targeting the PI3K/AKT pathway in cisplatin-resistant TNBC, our systems analysis found many differential interactions (Figure 1) and an elevated activity state (Figure S2D) of the AKT signalling axis, suggesting that inhibition of PI3Kalpha, PDK1, AKT-S473, and the AKT target p27kip should inhibit the growth of cisplatin-resistant TNBC cell lines. In our panel of TNBC cell lines, those with PIK3CA mutations had the greatest sensitivity to taselisib; however, all cell lines had IC_50_s < 1 μM. Again, the PIK3CA mutant MFM223 cells had the greatest sensitivity to both taselisib (PI3Kα/δ) and ipaterisib (AKTi).

Several studies have targeted the PIK3/AKT pathway in TNBC [29,30] with success. Supporting our hypothesis, however, Gohr et al. 2017 identified that the PI3K/AKT signalling pathway is associated with cisplatin resistance [31], and that as acquired resistance to cisplatin developed the levels of *p*-AKT in cancer cells also increased [31]. However, in contrast to our study, Gohr et al. only confirmed the impact of inhibiting PI3K/AKT signalling in models of acquired cisplatin resistance.

TNBC features frequent TP53 mutations, and previous studies have linked TP53 mutation status with increased activity of the PIK3CA gene and downstream PI3K-AKT signalling [32]. Astanehe et al. demonstrated that a loss of p53 function increases the expression of the PI3K p110 isoform in ovarian cancer [33], whilst Thakur et al. identified that ovarian cells that had nonfunctional TP53 and were cisplatin resistant exhibited higher phosphorylation of AKT [34]. Clinically, Chen et al. identified that in a population of 353 breast cancer patients, 18 (5%) had a co-mutation of TP53 and PIK3CA [35], and this population consisted of mainly cancers that were hormone-receptor negative. Finally, there is preclinical evidence to demonstrate the tumourigenic impact of PIK3CA/TP53 mutated cancers [36,37,38], and that this subset of breast cancers has a worse clinical prognosis [35].

Therefore, to confirm that targeting TNBC cell lines that harbour either or both TP53 and PIK3CA mutations is an effective treatment for cisplatin-resistant TNBC, we tested combinations of either PI3Ki or AKTi with cisplatin. We found that TNBC cell lines responded synergistically to the combination; however, the MFM223 cell lines which harbour a dual PIK3CA TP53 mutation and were cisplatin resistant responded best to both combinations.

Further assessing why this combination of drugs has robust anticancer effects in cisplatin-resistant TNBC cell lines; studies have associated that cell lines with PIK3CA mutations are associated with chemotherapy resistance [39]. Specifically, evidence demonstrates that PI3K/AKT pathway activation results in the increased expression of adenosine triphosphate (ATP)-binding cassette (ABC) transporters, which can result in multidrug resistance (MDR) [24]. As mentioned previously, MDR is a common resistance mechanism to intrinsic cisplatin resistance. Aberrations that result in the activation of the PI3K/AKT pathway in TNBC likely result in increased expression of MDR-related genes such as PgP, MDR1, and MDR2. This upregulation of MDR genes makes TNBC cells resistant to cisplatin and other chemotherapies. By testing the PI3K or AKT inhibitors, taselisib and ipatasertib, in TNBC, we were able to reduce PI3K/AKT signalling, which subsequently reduces MDR expression and enables cisplatin to effectively kill TNBC cells.

## 5. Conclusions

In conclusion, our results demonstrate that TP53- and PIK3CA-mutant TNBC cell lines with innate resistance to cisplatin are sensitive to drug combinations of cisplatin and PI3K/AKT pathway inhibitors, representing a potential therapeutic approach that warrants further investigation.

## Figures and Tables

**Table 1:** Small molecule inhibitors and concentrations used in the reverse-phase protein array analysis.

Target	Inhibitor	Concentration (nM)	Duration of Treatment
AKT	Ipatasertib	500	1 h
AMPK	AICAR (Acadesine) *	1	1 h
AMPK	Dorsomorphin **	20,000	1 h
RAF	TAK-632	20,000	1 h
FAK	PF-00562271	100	1 h
GSK3B	CHIR-98014	10,000	1 h
MEK	U0126	10,000	1 h
mTOR	Everolimus	1	1 h
NFkB	QNZ (EVP4593)	1000, 5000	1 h
p70S6K	PF-4708671	10,000	1 h
STAT3	Stattic	5000	1 h
DNA	Cisplatin	5000	1 h

Inhibitors were chosen to target key nodes in breast cancer signalling pathways. * AICAR is an AMPK activator; ** also inhibits activin receptor-like kinases ALK2/3/6.

**Table 2:** Cisplatin sensitivity in a panel of TNBC cell lines as determined by the acid phosphatase assay.

Cell Lines	Triple Negative Subtype	Cisplatin IC50 (µM)	Cisplatin (% Inhibition @ 10 µM)	Cisplatin Response
HDQ-P1	Basal-like 2	6.33 ± 0.46	53.4 ± 2.3	Resistant
MDA-MB-157	Mesenchymal stem-like	8.90 ± 0.83	47.8 ± 16.1	Resistant
MFM223	LAR	>10	23.3 ± 2.1	Resistant
CAL120	Mesenchymal-like	12.3 ± 3.6	44.6 ± 2.3	Resistant
MCF10A	Normal	5.25 ± 0.55	69.5 ± 2.6	-
MDA-MB-468	Basal-like 1	0.23 ± 0.03	99.5 ± 0.5	Sensitive
HCC1143	Basal-like 1	1.07 ± 0.32	95.9 ± 2.1	Sensitive
MDA-MB-231	Mesenchymal stem-like	2.13 ± 0.42	77.5 ± 8.4	Sensitive
CAL-85-1	Basal-like 2	0.47 ± 0.04	96.6 ± 1.0	Sensitive

Standard deviations are calculated from triplicate independent assays. Cisplatin response in TNBC cell lines is determined relative to the cisplatin sensitivity of the MCF10A cell line. IC50 values were calculated using CalcuSyn software ™.

**Table 3:** Sensitivity of a panel of TNBC cell lines as determined by the acid phosphatase assay to cisplatin, an S6-kinase; a GSK3B; a PI3K; and an AKT small-molecule inhibitor. Standard deviations are calculated from triplicate independent assays using the CalcuSyn software ™. Mutational status of TP53 and PIK3CA were obtained from the Cancer Cell Line Encyclopaedia (https://portals.broadinstitute.org/ccle accessed on 15 February 2022).

Cell Line	PIK3CA Mutation	TP53 Mutation	Cisplatin IC_50_ μM	M2698 IC_50_ μM	CHIR-98014 % Inhibition @ 1 μM	Taselisib IC_50_ μM	Ipataseritib IC_50_ μM
			Cisplatin	P70S6 Kinase Inhibitor	GSK3B Inhibitor	PI3K Inhibitor (αδ Specific)	AKT Inhibitor
BT20	H1047R/P539R	K132Q	0.43 ± 0.08	n/a	n/a	0.11 ± 0.03	n/a
CAL-51	E542K	WT	2.10 ± 0.60	n/a	n/a	0.03 ± 0.02	n/a
CAL85-1	WT	K132E	0.47 ± 0.04	0.94 ± 0.65	n/a	n/a	n/a
HCC1143	WT	R248Q	1.07 ± 0.32	1.90 ± 0.58	−5 ± 6%	n/a	n/a
HDQ-P1	WT	R213 *	6.33 ± 0.46	7.34 ± 3.40	37 ± 10%	0.67 ± 0.16	n/a
MDA-MB-157	WT	FS Del	8.90 ± 0.83	4.11 ± 1.11	20 ± 21%	>5 uM	n/a
MDA-MB-231	WT	R280K	2.13 ± 0.42	2.00 ± 0.45	49 ± 4%	n/a	n/a
MDA-MB-468	WT	R273H	0.23 ± 0.03	0.38 ± 0.91	82 ± 7%	n/a	n/a
MFM-223	H1047R	K132R	>10	0.11 ± 0.02	17% ± 6	0.17 ± 0.06	0.27 ± 0.15

* Indicates a stop mutation.

**Table 4:** Cisplatin-resistant TNBC cell lines that are both PIK3CA and TP53 mutant respond best to the combination of taselisib (PI3K inhibitor) and cisplatin. Further, the MFM-223 cisplatin-resistant TNBC cell lines that is PIK3CA and TP53 mutant has the best response to the combination of ipatasertib (AKTi) and cisplatin. TNBC cells with innate resistance to cisplatin are highlighted in red, whilst TNBC cells with innate sensitivity to ciaplatin are highlighted in green. Standard deviations are calculated from triplicate independent assays. Loewe synergy calculated using Combifit Analysis ™. IC_50_ results calculated using the CalcuSyn software ™. Mutational status of TP53 and PIK3CA were obtained from the Cancer Cell Line Encyclopaedia (https://portals.broadinstitute.org/ccle accessed on 15 February 2022).

Cisplatin ± Taselisib-PI3K Inhibitor						
Cell Line	Cisplatin IC_50_ (μM)	PIK3CA Mutation	TP53 Mutation	Best Loewe Synergy	PI3K Inhi#bitor conc. (nM)	Cisplatin (nM)
MFM223	>10	H1047R	K132R	21 (±6)	75	5000
HDQP1	6.4	WT	R213 *	9 (±3)	25	1250
MDAMB157	8	WT	FS Del	13 (±5)	100	1250
CAL51	2.1	E542K	WT	12 (±2)	5	600
BT20	0.43	H1047R/ P539R	K132Q	11 (±2)	150	200
**Cisplatin ± Ipatasertib-AKT Inhibitor**						
Cell Line	Cisplatin IC_50_ (μM)	PIK3CA Mutation	TP53 mutation	Best Loewe synergy	AKTi conc. (nM)	Cisplatin (nM)
MFM223	>10	H1047R	K132R	19 (±4)	75	5000

* Indicates a stop mutation.

## Data Availability

The code for the computational analysis, including the raw and processed data are available in github: https://github.com/dirkfey/TNBC-RPPA (accessed on 15 February 2022).

## Supplementary Materials

The following supporting information can be downloaded at: https://www.mdpi.com/article/10.3390/jpm12081277/s1, Supplementary Materials and Methods: List of antibodies analysed on the reverse-phase protein array platform. Supplementary Figure S1: Hierarchical clustergrams of treatment-induced signalling changes for each drug. Supplementary Figure S1B: Responsiveness of the cell lines in terms of their drug-induced changes in network nodes. Supplementary Figure S2A: Inferred interaction networks for each cell line as indicated. Supplementary Figure S2B,C: (B) Consensus networks for sensitive and resistant cells. (C) Table of the most significant interaction differences between sensitive and resistant cells. Supplementary Figure S2D: The most significant differences in the baseline network state in the unperturbed condition. Supplementary Figure S2E–J: Analysis of METABRIC data. Supplementary Figure S3: Growth inhibitory effect of cisplatin in combination with taselisib (PI3K inhibitor) in the (A) HDQP1, (B) MDAMB157, (C) CAL51, and (D) BT20 breast cancer cell lines.
