# Supplementary material for: A Systems Biology Approach to Investigate Kinase Signal Transduction Networks That Are Involved in Triple Negative Breast Cancer Resistance to Cisplatin
Source: J Pers Med. 2022 Aug 3;12(8):1277. doi: 10.3390/jpm12081277 (PMC9409860; doi:10.3390/jpm12081277)
Supplement: Supplementary file 1 [file jpm-12-01277-s001.zip › jpm-1719903-supplementary.pdf]

Supplementary Materials and Methods: List of antibodies analysed on the Reverse Phase Protein Array platform.

Antibody	Cat#	Company	Dil	Host
AKT (S473)	9271	CellSig	1:250	R
AKT (T308)	2965	CellSig	1:500	R
Akt2	2964	CellSig	1:50	R
AMPK (T172)	2535	CellSig	1:250	R
AMPKalpha1	2532	CellSig	1:250	R
Bcl-2	MO887	Dako	1:250	R
Bcl-2 (S70)	2827	CellSig	1:100	R
Bcl-2 (T56)	2875	CellSig	1:100	R
Caspase 8	9746	CellSig	1:1000	M
Caspase-7, cleaved (D198)	9491	CellSig	1:100	R
Caspase-9, cleaved (D315)	9505	CellSig	1:100	R
Caspase-9, cleaved (D330)	9501	CellSig	1:100	R
Chk-1	2345	CellSig	1:250	R
Chk-1 (S345)	2348	CellSig	1:50	R
C-Raf	04-739	Millipore	1:250	R
c-Raf (S338)	9427	CellSig	1:200	R
EGFR	2232	CellSig	1:100	R
FAK	1700-1	Epitomics	1:500	R
FAK (Y925)	3284	CellSig	1:4000	R
Gab1	3232	CellSig	1:200	R
Gab1 (Y627)	3233	CellSig	1:500	R
GSK-3β	610201	BD	1:300	M
GSK-3β (S9)	9336	CellSig	1:500	R
HER2	MS-325-P1	Lab Vision	1:1000	M
HER2 (Y1248)	06-229	Upstate	1:750	R
HIAP-2 (cIAP-1)	07-759	Millipore	1:250	R
IGFIR-Beta	3027	CellSig	1:500	R
MAPK - ERK 1/2	9102	CellSig	1:200	R
MAPK (T202/Y204) -ERK1/2	4377	CellSig	1:1200	R
MEK1	1235-1	Epitomics	1:1200	R
MEK1/2 (S217/221)	9154	CellSig	1:1000	R
mTOR	2983	CellSig	1:400	R
mTOR (S2448)	2971	CellSig	1:100	R
mTOR (S2481)	2974	CellSig	1:100	R
NF-kB-p65 (S536)	3033	CellSig	1:100	R

p27	1591-1	Epitomics	1:250	R
p27 (T157)	AF1555	R&D	1:150	R
p38 MAP Kinase (T180/Y182)	9211	CellSig	1:250	R
p38_MAPK	9212	CellSig	1:300	R
p53	9282	CellSig	1:3000	R
p70 S6 Kinase	1494-1	Epitomics	1:250	R
p70 S6 Kinase (T389)	9205	CellSig	1:250	R
PDK1	3062	CellSig	1:100	R
PDK1 (S241)	3061	CellSig	1:500	R
PI3-Kinase p110alpha	4255	CellSig	1:100	R
PKC-alpha	05-154	Upstate	1:2000	M
PTEN	9552	CellSig	1:1000	R
S6 Ribosomal Protein (S235/236) (2F9)	4856	CellSig	1:200	R
S6 Ribosomal Protein (S240/244)	2215	CellSig	1:3000	R
Smac/Diablo	2954	CellSig	1:500	M
Src	05-184	Upstate	1:200	M
Src (Y527)	2105	CellSig	1:400	R
Src Family (Y416)	2101	CellSig	1:250	R
Stat3	9132	CellSig	1:1000	R
Stat3 (Y705)	9131	CellSig	1:500	R
Bak	ab32371	Abcam	1:200	R
Bax	2772	CellSig	1:250	R
Bcl-xL	2762	CellSig	1:250	R
Mcl-1	5453	CellSig	1:1000	R
Apaf-1	HPA031373	Sigma	1:100	R
Cytochrome C	4280	CellSig	1:2000	R
Caspase 3	9662	CellSig	1:5000	R
Caspase 9	9502	CellSig	1:1000	R
cMET	3127	CellSig	1:500	M
cMET Y1234/Y1235	3129	CellSig	1:100	R
RSK	9347	CellSig	1:500	R
P90RSK S380	9341	CellSig	1:250	R
EGFR Y992	2235	CellSig	1:100	R
EGFR Y1068	2234	CellSig	1:100	R
lhda	3582	CellSig	1:200	R
tigar	137573	AbCam	1:100	R
her4	4795	CellSig	1:100	M

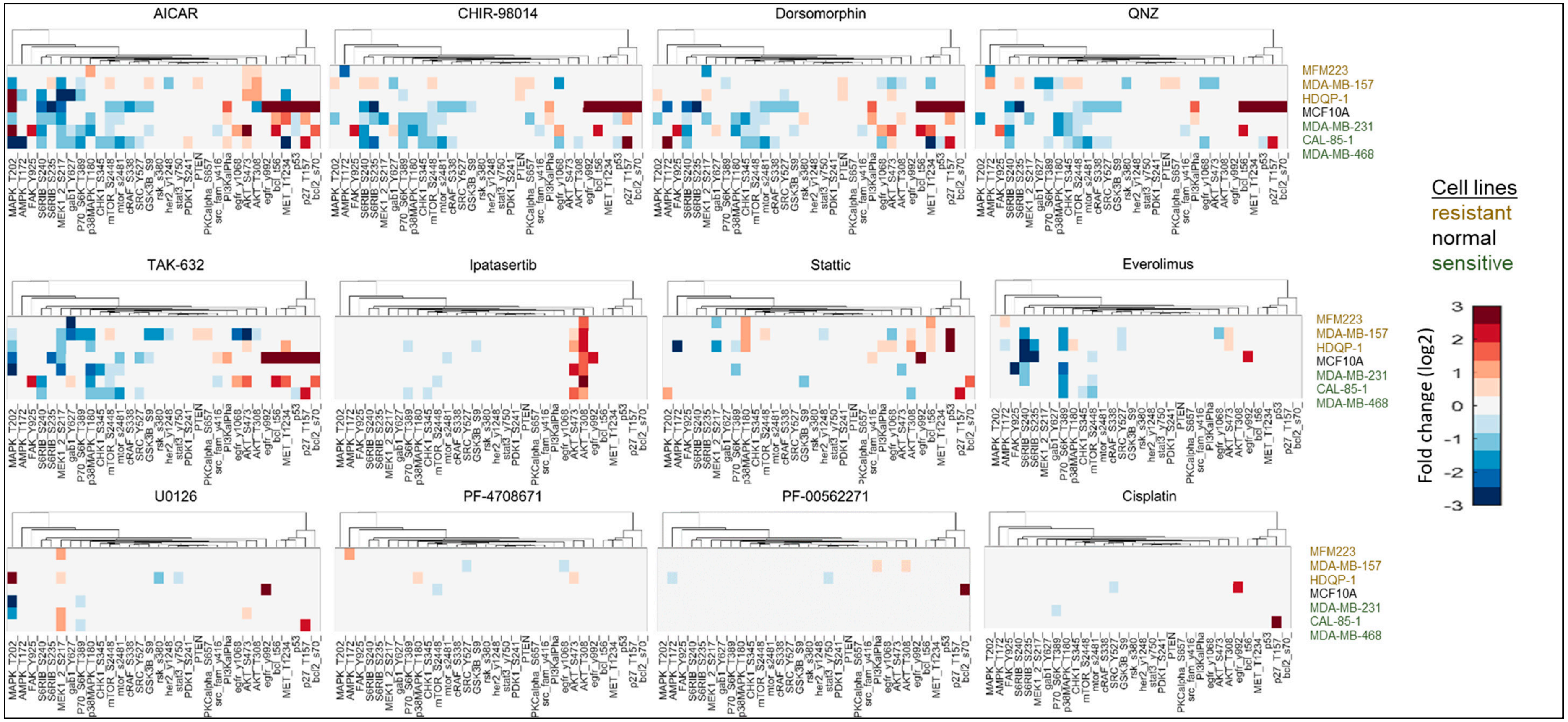

Supplementary Figure S1. Hierarchical clustergrams of treatment induced signaling changes for each drug. Top three rows: resistant cell lines, bottom three rows: sensitive cell lines.

(B1) (B2)

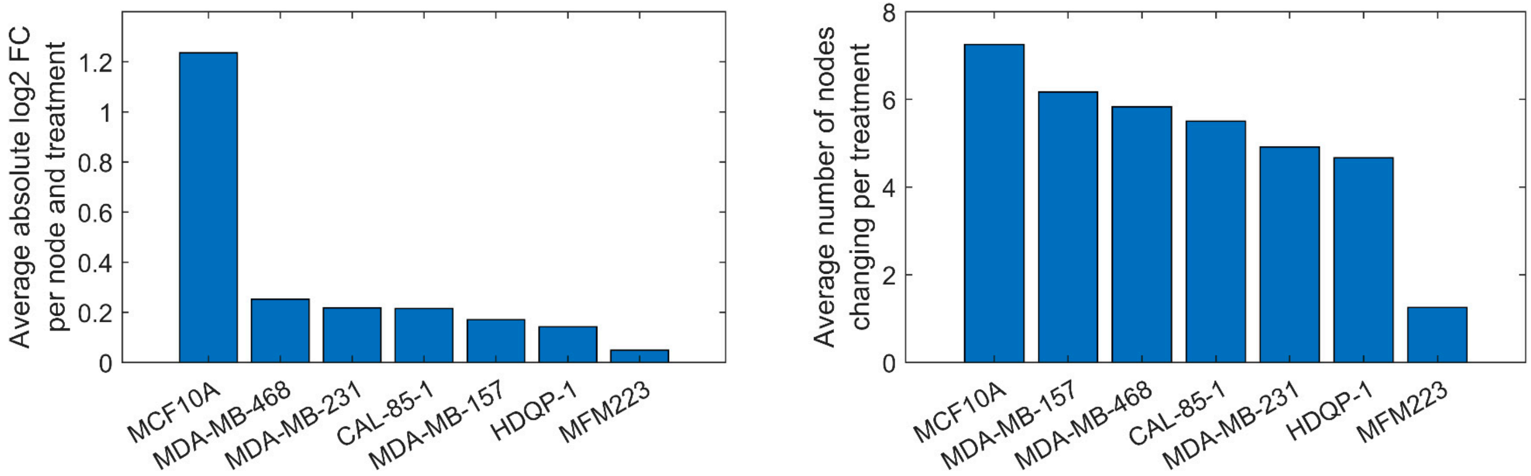

Supplementary Figure S1B: Responsiveness of the cell lines in terms of their drug induced changes in network nodes. (B1) Average mean fold-change of each cell line defined as the fold-changes of the network node averaged over all drugs and network nodes. (B2) Average number of nodes changing per drug (cut-off Students t-test p-value < 0.05).

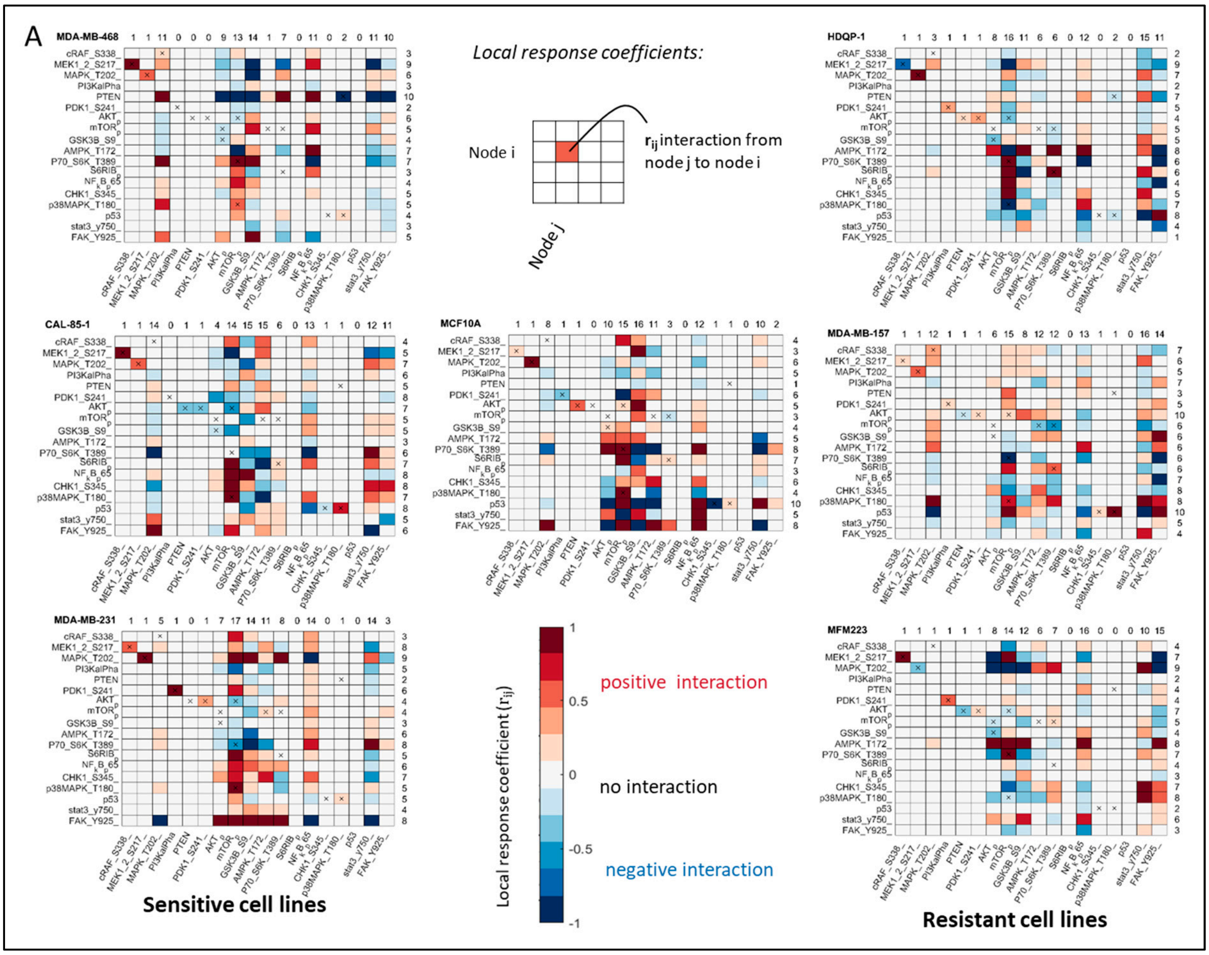

Supplementary Figure S2A: Inferred interaction networks for each cell line as indicated. Colors denote the BMRI estimates of the local response coefficients; red – positive, blue – negative, x-marks indicate canonical interactions of the prior network.

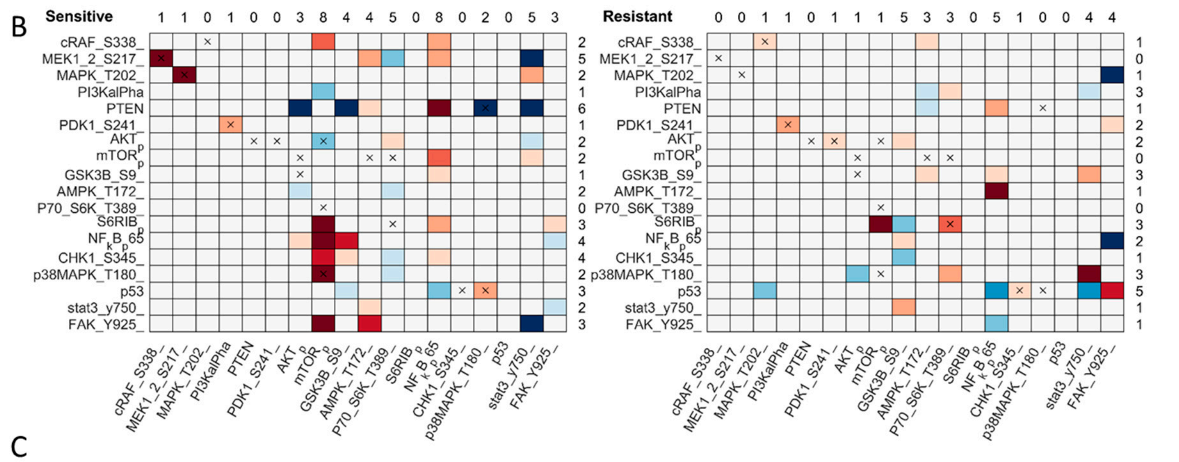

C

Local response coefficients Interaction	pvalue*	Delta**	Sensitive cell lines MDA_MB_468	CAL_85_1	MDA_MB_231	Resistant cell lines HDQP_1	MDA_MB_157	MFM223
AKT_p -> p38MAPK_T180_	0.003	-0.227	-0.083	-0.016	-0.082	-0.267	-0.252	-0.342
GSK3B_S9_ -> CHK1_S345_	0.005	-0.514	0.093	0.325	0.140	-0.430	-0.219	-0.334
AMPK_T172_ -> PTEN	0.032	-0.297	0.237	0.334	0.032	-0.144	-0.068	-0.077
AKT_p -> PI3KalPha	0.032	0.119	-0.050	-0.052	-0.110	-0.008	0.100	0.052
FAK_Y925_ -> PDK1_S241_	0.040	0.278	-0.085	-0.150	-0.011	0.032	0.305	0.249
FAK_Y925_ -> p53	0.050	0.832	-0.153	0.017	-0.003	1.275	0.827	0.255
mTOR_p -> cRAF_S338_	0.053	-0.682	0.252	0.500	0.792	-0.143	0.159	-0.518
NF_kB_p65 -> cRAF_S338_	0.058	-0.287	0.202	0.419	0.301	0.004	-0.126	0.182

*2 sample Students t-test (sensitive versus resistant)
**mean(resistant) - mean(sensitive)

Supplementary Figure S2B, C: (B) Consensus networks for sensitive and resistant cell lines. The consensus network was calculated by filtering out inconsistent interactions; we kept only interactions for which the estimated local response coefficient rij were either all positive or all negative for the set of sensitive and resistant cell line. The consensus interactions strength was calculated as the mean r : rij,consensus = mean(rij,celllines), where celllines is the set of sensitive (left) and resistant (right) cell lines. The numbers above the rows and next to the columns indicate the number of non-zero interactions. (C) Table of the most significant interaction differences between sensitive and resistant cell lines. Negative values shaded blue indicate inhibition; positive values shaded red indicate activation.

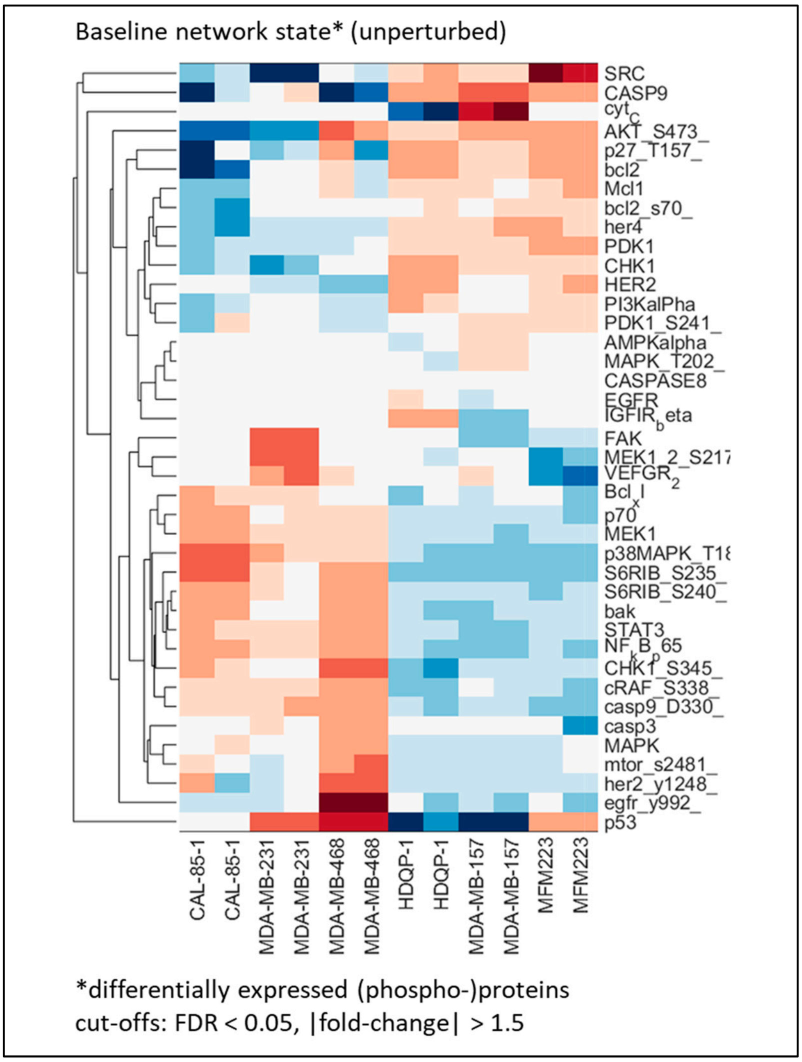

Supplementary Figure S2D: The most significant differences in the baseline network state in the unperturbed condition. The heatmap shoes the hierarchical clustering was performed of the most significant changes. Cut-offs were false discovery rate FDR<0.05, absolute fold change >1.5. Green arrows indicate analytes of the PI3K-AKT signaling axis.

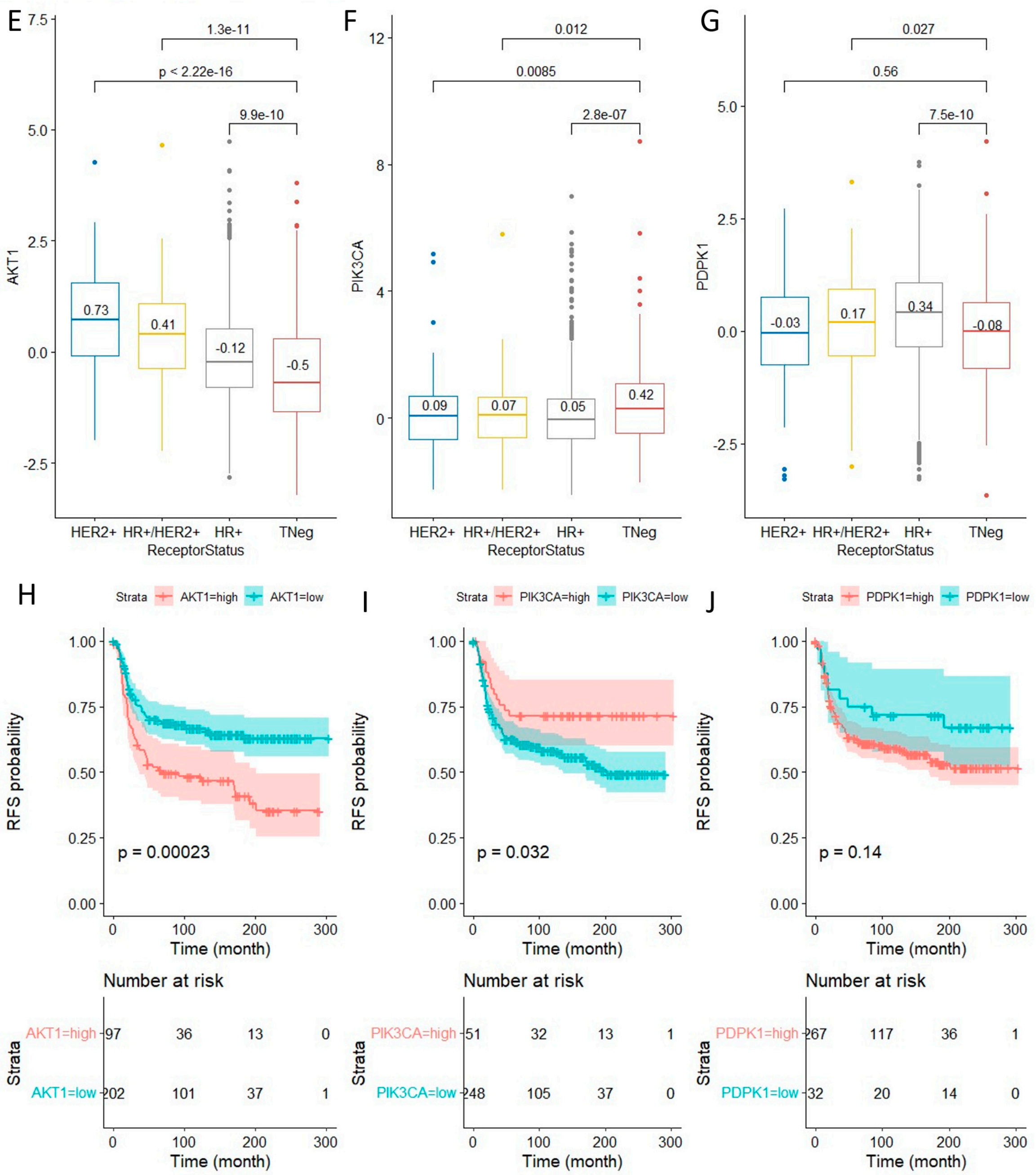

Supplementary Figure S2E-J Analysis of METABRIC data. (E-G) Boxplots of gene-expression distributions for the different subtypes as indicated; (E) AKT1, (F) PIK3CA, (G) PDPK1. Numbers in boxes indicate mean value of mRNA expression (log2), p-values on top of comparison bars from Wilcoxon rank-sum test. (H-J) Kaplan-Meier survival analysis based on the expression of each gene as indicated; (H) AKT1, (I) PIK3CA, (J) PDPK1. RFS: relapse free survival, p: p-value from logrank test, shaded area: 95% confidence interval.

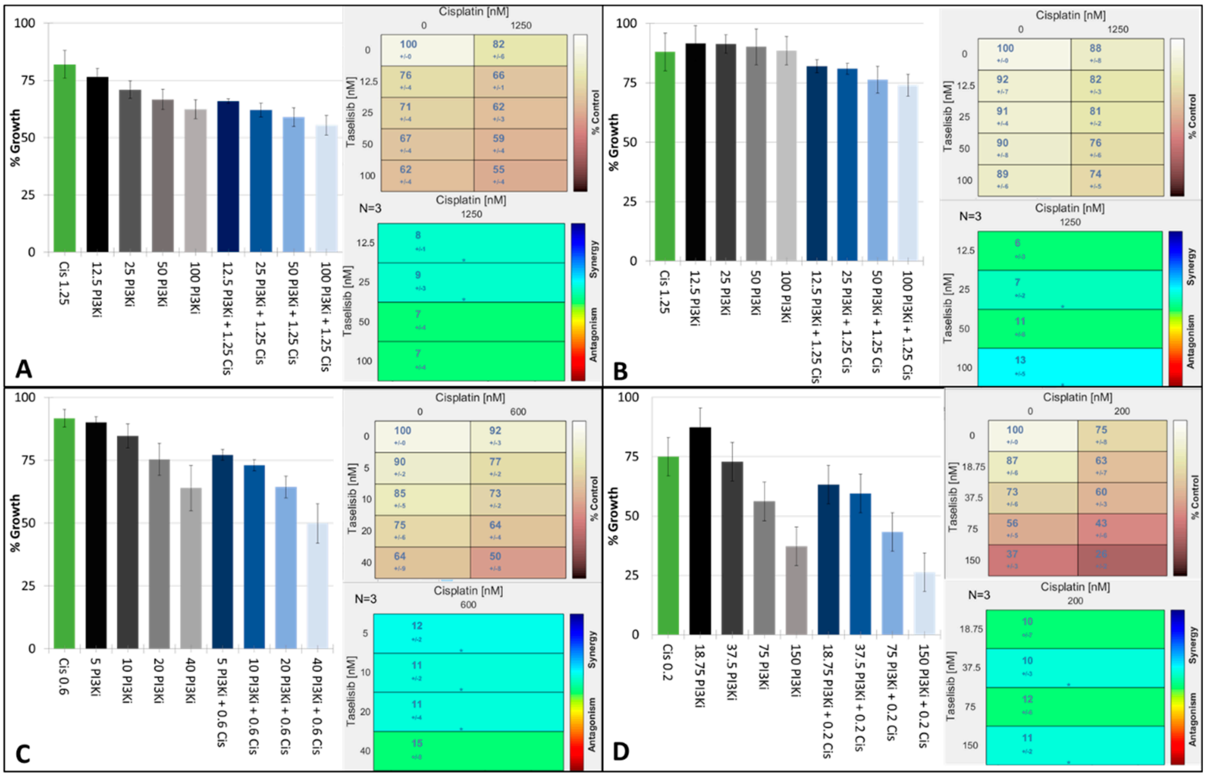

Supplementary Figure S3: Growth inhibitory effect of cisplatin in combination with Taselisib (PI3K inhibitor) in the (A) HDQP1 (B) MDAMB157 (C) CAL51 (D) BT20 breast cancer cell lines. Standard deviations are calculated from triplicate independent assays. Combifit analysis calculates the average % growth of either cisplatin or taselisib tested alone or in combination, whilst Loewe analysis calculates the synergy of the combination.
